# Supplementary material for: Developmental and Nutritional Changes in Children with Severe Acute Malnutrition Provided with n-3 Fatty Acids Improved Ready-to-Use Therapeutic Food and Psychosocial Support: A Pilot Study in Tanzania
Source: Nutrients. 2024 Feb 28;16(5):692. doi: 10.3390/nu16050692 (PMC10934689; doi:10.3390/nu16050692)
Supplement: Supplementary file 1 [file nutrients-16-00692-s001.zip › nutrients-2826587-supplementary.pdf]

Figure S1. Flowchart of phases and processes of work undertaken in the BrightSAM feasibility and pilot study.

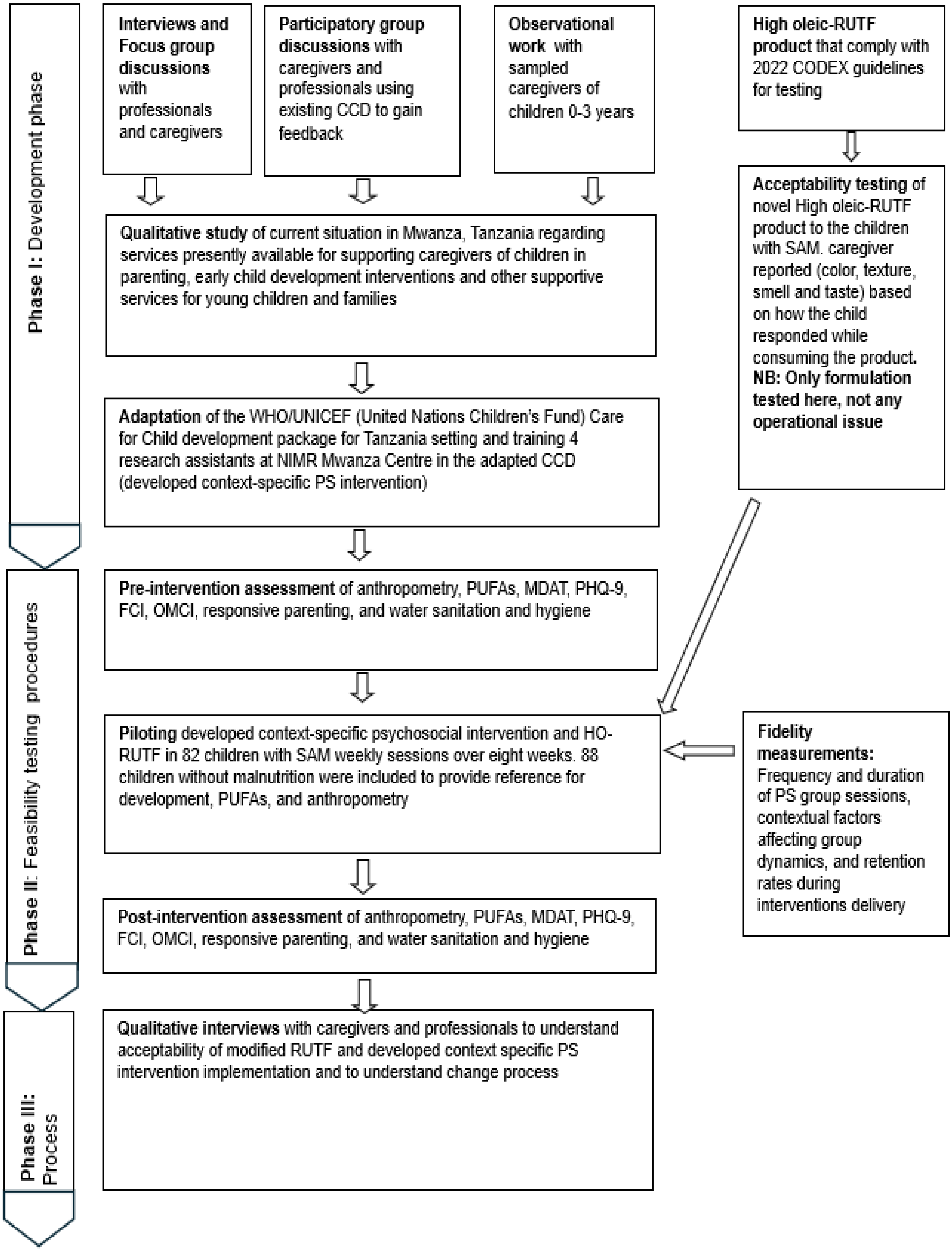

Table S1: Nutritional composition of ready-to-use therapeutic food

	**For 100g of RUTF EFA+**
	**Mean value**
**Macronutrient**	
Energy, kcal	543
Protein, g	14.1
Protein, dairy, g	0.84
Lipids, g	33.8
Linolenic acid, g	3.7
alpha linoleic acid, g	1.02
Carbohydrates, mg	44.5
Total fibres	1.6
Moisture, g	0
DHA, mg	0
EPA, mg	0
**Micronutrient**	
Phosphorus, mg	384
of which free phosphorus, mg	340
Calcium, mg	358
Potassium, mg	1374
Magnesium, mg	88
Zinc, mg	12.8
Copper, mg	1.7
Iron, mg	11.4
Iodine, µg	123
Selenium, µg	38
Sodium, mg	166
Vitamin A, mg RE	0.86
Vitamin D, µg	15
Vitamin E, mg alpha-TE	20
Vitamin C, mg	50
Vitamin B1, mg	0.54
Vitamin B2, mg	1.6
Vitamin B6, mg	0.6
Vitamin B12, µg	1.6
Vitamin K, µg	28
Biotin, µg	64
Folic acid, µg	200
Pantothenic acid, mg	3
Niacin, mg	5

Table S2: Family care indicators, caregiver-child interaction, and maternal depression in children with and without severe acute malnutrition

	Baseline			Frequencies at 8 weeks
	SAM, n=70 N (%)	Non-SAM, n=78 N (%)	^{1}P	SAM, n=70 N (%)
**Household books**			0.24	
None	62 (89)	61 (78)		50 (71)
1-2	7 (10)	15 (19)		18 (26)
3-4	1 (1)	2 (3)		2 (3)
**Sources of play materials**				
Home-made toys	0 (0)	3 (4)	0.10	51 (73)
Purchased toys	25 (36)	39 (50)	0.08	28 (40)
Household objects	67 (96)	78 (100)	0.07	67 (96)
**Variety of play materials**				
Things that make/play music	8 (11)	14 (18)	0.27	18 (26)
Things for drawing/writing	14 (20)	27 (35)	0.05	23 (33)
Children's picture books	1 (1)	8 (10)	0.03	10 (14)
Things for stacking/construction/building	7 (10)	15 (19)	0.12	8 (11)
Things for moving around (eg. toy bicycle)	1 (1)	3 (4)	0.35	2 (3)
Toys for learning shapes/colours	0 (0)	2 (3)	0.18	2 (3)
Things for pretending (eg. cooking or shopping)	5 (7)	18 (23)	0.008	9 (13)
**Family interaction**				
Read books or looked at picture books	3 (4)	12 (15)	0.03	18 (26)
Tell stories	6 (9)	7 (9)	0.93	12 (17)
Sing songs	58 (83)	70 (90)	0.22	70 (100)
Take the child outside the home	42 (60)	63 (81)	0.005	68 (97)
Played with the child	63 (90)	77 (99)	0.02	70 (100)
Spend time naming things/counting/drawing	16 (23)	33 (42)	0.02	32 (46)
**Maternal depression scale (PHQ9)**			<0.001	
Non/minimal	22 (31.4)	56 (71.8)		63 (90.0)
Mild	15 (21.4)	9 (11.5)		3 (4.3)
Moderate	17 (24.3)	4 (5.1)		2 (2.9)
Moderately severe	9 (12.9)	4 (5.1)		1 (1.4)
Severe	7 (10.0)	5 (6.4)		1 (1.4)

[1]p= p-value (chi-squared test) for difference between children with and without SAM at baseline. PHQ9= Patient Health Questionnaire-9. SAM= severe acute malnutrition
